# FtsH2-Dependent Proteolysis of EXECUTER1 Is Essential in Mediating Singlet Oxygen-Triggered Retrograde Signaling in *Arabidopsis thaliana*

**DOI:** 10.3389/fpls.2017.01145

**Published:** 2017-06-29

**Authors:** Vivek Dogra, Jianli Duan, Keun Pyo Lee, Shanshan Lv, Renyi Liu, Chanhong Kim

**Affiliations:** ^1^Laboratory of Photosynthesis and Stress Signaling, Center for Excellence in Molecular Plant Sciences, Shanghai Center for Plant Stress Biology, Chinese Academy of SciencesShanghai, China; ^2^Shanghai Center for Plant Stress Biology, Chinese Academy of SciencesShanghai, China

**Keywords:** ^1^O_2_, retrograde signaling, EXECUTER 1, FtsH2 protease, β-carotene, β-cyclocitral, chloroplast

## Abstract

Photosystem II reaction center (PSII RC) and light-harvesting complex inevitably generate highly reactive singlet oxygen (^1^O_2_) that can impose photo-oxidative damage, especially when the rate of generation exceeds the rate of detoxification. Besides being toxic, ^1^O_2_ has also been ascribed to trigger retrograde signaling, which leads to nuclear gene expression changes. Two distinctive molecular components appear to regulate ^1^O_2_ signaling: a volatile signaling molecule β-cyclocitral (β-CC) generated upon oxidation of β-carotene by ^1^O_2_ in PSII RC assembled in grana core, and a thylakoid membrane-bound FtsH2 metalloprotease that promotes ^1^O_2_-triggered signaling through the proteolysis of EXECUTER1 (EX1) proteins associated with PSII in grana margin. The role of FtsH2 protease in ^1^O_2_ signaling was established recently in the conditional *fluorescent* (*flu*) mutant of *Arabidopsis thaliana* that generates ^1^O_2_ upon dark-to-light shift. The *flu* mutant lacking functional FtsH2 significantly impairs ^1^O_2_-triggered and EX1-mediated cell death. In the present study, the role of FtsH2 in the induction of ^1^O_2_ signaling was further clarified by analyzing the FtsH2-dependent nuclear gene expression changes in the *flu* mutant. Genome-wide transcriptome analysis showed that the inactivation of FtsH2 repressed the majority (85%) of the EX1-dependent ^1^O_2_-responsive genes (SORGs), providing direct connection between FtsH2-mediated EX1 degradation and ^1^O_2_-triggered gene expression changes. Furthermore, the overlap between β-CC-induced genes and EX1-FtsH2-dependent genes was very limited, further supporting the coexistence of two distinctive ^1^O_2_ signaling pathways.

## Background

Under a multitude of environmental factors, altered levels of reactive oxygen species (ROS) in chloroplasts, which have long been implicated with damaging of macromolecules, appear to trigger certain signaling cascades leading to nuclear gene expression changes via a process known as retrograde signaling (Apel and Hirt, 2004). The transcriptional reprogramming seems to be essential for plants to sustain and adapt to the environmental changes. Among various ROS, chloroplasts generate ^1^O_2_ during oxygenic photosynthesis at the active PSII RC residing in the grana core (appressed grana region) of the thylakoids (Triantaphylidès et al., 2008). In order to minimize the photo-oxidative damage caused by ^1^O_2_, plants utilize molecular components residing in PSII RC, such as β-carotene and D1 protein, to quench ^1^O_2_ (Triantaphylidès et al., 2008). Oxidation of D1 protein leads to PSII damage, which subsequently undergoes disassembly and reassembly processes through the proteolysis of the damaged D1 proteins by ATP-dependent hexameric FtsH metalloprotease and the concurrent *de novo* synthesis of D1 proteins, respectively (Kato and Sakamoto, 2009; Kato et al., 2012).

^1^O_2_ has also been reported to trigger chloroplast-to-nucleus retrograde signaling, which is manifested by altered nuclear gene expression, acclimation response and programmed cell death (PCD) (op den Camp et al., 2003; Wagner et al., 2004; Lee et al., 2007; Kim et al., 2008, 2012; Ramel et al., 2012, 2013; Wang et al., 2016). Because ^1^O_2_ has an extremely short lifespan (∼200 ns) in a cellular environment (Li et al., 2012; Telfer, 2014), it is unlikely to serve as a signaling molecule. Therefore, chloroplasts may contain ^1^O_2_ sensor(s) to translate the levels of ^1^O_2_, either by self-oxidation or by monitoring the oxidation of other molecule(s), to subsequent changes in cellular processes. As ^1^O_2_ is generated in PSII, it is reasonable to assume that ^1^O_2_ sensor might be physically associated with PSII. Accordingly, the PSII components, such as D1 protein and β-carotene have long been supposed to act as primary scavengers of ^1^O_2_ (Triantaphylidès and Havaux, 2009). Even though the degradation product of D1 has been shown to control the synthesis of D1 in cyanobacteria (Stelljes and Koenig, 2007), there is no evidence to support that the degradation product of D1 acts as a signaling molecule in plants.

In contrast, strong evidence has been found to support that β-carotene, a primary quencher of ^1^O_2_ and a constituent of PSII RC, is a ^1^O_2_ sensor. β-cyclocitral (β-CC), a volatile oxidized derivatives of β-carotene, has been found to act as a signaling molecule in Arabidopsis wild-type plants exposed to excess light stress (Ramel et al., 2013). Moreover, plants treated with β-CC upregulate a set of ^1^O_2_-responsive genes (SORGs) (Ramel et al., 2012) identified previously using conditional Arabidopsis *flu* mutant generating ^1^O_2_ upon dark-to-light shift (op den Camp et al., 2003). In addition, β-CC has also been implicated with the acclimation responses in *chlorina 1* (*ch1*) mutant lacking light-harvesting complex (LHC) of PSII, in which the level of ^1^O_2_ under light stress was escalated (Ramel et al., 2013). Although the volatile can diffuse freely into the nucleus, the molecular mechanism underlying the β-CC-mediated transcriptional reprogramming is largely unknown. Nonetheless, the earlier genetic screen using the unicellular green alga *Chlamydomonas reinhardtii* uncovered Methylene Blue Sensitivity 1 (MBS1), a small Zinc finger protein, as a mediator of ^1^O_2_ signaling (Shao et al., 2013). While the MBS1 protein is present in both cytosol and nucleus under normal conditions, it concentrates in specific stress granules and processing bodies under oxidative stress (Shao et al., 2013). A recent study revealed that MBS1 was upregulated in wild-type Arabidopsis plants under β-CC treatment even in normal light conditions, which led to gene expression changes for photo-acclimation (Shumbe et al., 2017). However, a further study is needed to show whether MBS1 can directly bind to DNA or requires other mediators to alter the nuclear gene expression.

Another distinct ^1^O_2_ signaling has been identified earlier in Arabidopsis *flu* mutant (op den Camp et al., 2003). In the dark, FLU protein acts as a negative feedback regulator of tetrapyrrole synthesis in Mg^2+^-branch. As the reduction of protochlorophyllide (Pchlide) to chlorophyllide is catalyzed by light-dependent enzyme protochlorophyllide oxidoreductase (POR), the *flu* mutant overaccumulates Pchlide in the dark. As a potent photosensitizer, the accumulated Pchlide upon illumination absorbs light energy and subsequently transfers the absorbed light energy to stable molecular oxygen, resulting in the formation of highly reactive ^1^O_2_ in chloroplasts (Meskauskiene et al., 2001). The *flu* mutant, therefore, offers a conditional system that allows generating ^1^O_2_ in a controlled and non-invasive manner (op den Camp et al., 2003). Various studies using the *flu* mutant revealed that the chloroplast-generated ^1^O_2_ can induce the nuclear gene expression changes and PCD (op den Camp et al., 2003; Lee et al., 2007). The previous genetic screen using *flu* mutant has revealed that nuclear-encoded and chloroplast-targeted protein EX1 mediates the ^1^O_2_-triggered stress responses (Wagner et al., 2004). Inactivation of EX1 significantly abolishes the ^1^O_2_-induced cell death and nuclear gene expression changes in *flu* mutant (Wagner et al., 2004; Lee et al., 2007). Besides EX1, its close homolog EXECUTER2 (EX2) has also been implicated with mediating ^1^O_2_ signaling. While inactivation of EX1 significantly attenuates the SORGs, inactivation of both EX1 and EX2 leads to almost complete repression of SORGs (Lee et al., 2007). In addition, EX1 and EX2 also appear to regulate the local and systemic gene expression changes in response to high light stress (Carmody et al., 2016).

Initially, it was contemplated that the two signaling cascades might be dependent on each other. However, in *ch1* mutant the β-CC was found to relay the signal under photoinhibitory conditions in an EX1-independent manner (Ramel et al., 2012), implying that the two signaling cascades might be independent. Considering that both signaling events are instigated from PSII, it is obscure how these two signaling events are triggered independently. This seeming paradox was resolved in our recent study in which EX1 proteins were found mainly in ‘grana margin’ where the repair of PSII and the chlorophyll synthesis happen, implying that EX1-mediated ^1^O_2_ signaling is initiated in grana margin rather than the grana core (Wang et al., 2016). The study has also shown that light-adapted *flu* plants accumulated Pchlide in the dark almost evenly in different fractions across the thylakoid membrane, such as grana core, grana margin, and stroma lamellae (Wang et al., 2016). Given that *ex1/flu* abrogates the ^1^O_2_-triggered stress responses including growth inhibition and cell death, ^1^O_2_ generated in either grana core or stroma lamellae may not irreversibly damage the photosynthetic apparatus.

Protein immunoprecipitation (IP) coupled with mass spectrometry (MS) unveiled a group of proteins potentially forming the EXECUTER complex in grana margin, including enzymes involved in chlorophyll biosynthesis, PSII RC proteins and FtsH proteases (Wang et al., 2016). Because EX1 is associated with PSII and FtsH protease, upon ^1^O_2_ generation, EX1 possibly undergoes similar oxidative post-translational modification as PSII RC proteins including D1 and CP43 (Dreaden et al., 2012). If the oxidation occurs in EX1, the modified EX1 by ^1^O_2_ may become a target of FtsH protease, which is shown to be the case of D1 (Kato and Sakamoto, 2009). This proteolysis of EX1 may lead to the release of a signal that might be a part of EX1 complex or EX1 itself. Indeed, FtsH2-dependent proteolysis of EX1 in response to ^1^O_2_ and the concurrent induction of cell death are shown to be attenuated in the FtsH2-defecient *flu* mutant, namely *var2*/*flu*, implying that the proteolysis of EX1 is crucial for initiating the retrograde signaling (Wang et al., 2016). To investigate the underlying molecular mechanisms, we examined the effect of EX1 proteolysis on the induction of SORGs in *flu* mutant.

## Experimental Results

### Identification of a True Set of SORGs

**FIGURE 1:**
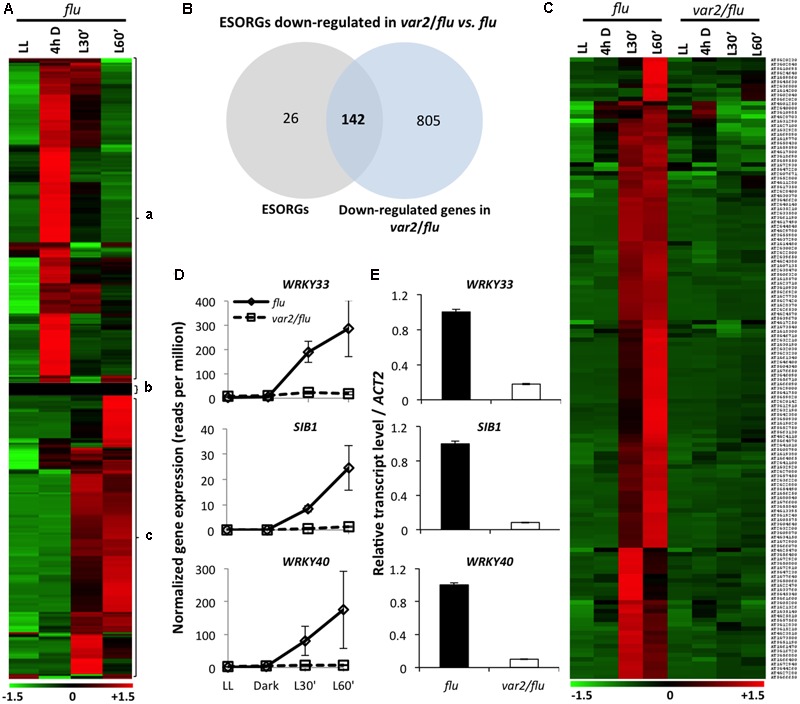
FtsH2 is an integral component of EX1-mediated ^1^O_2_ signaling. RNA- seq-based comparison of gene expression was performed in 5-day-old *var2/flu* and *flu* seedlings that were initially grown under continuous light (LL), transferred to dark for 4 hours (4 h D), and then re-exposed to light for 30 (L30′) and 60 min (L60′). **(A)** Heat map reclassified the expression of previously reported SORGs. After excluding 215 genes induced during dark incubation (a) and 26 genes that either exhibited no expression or were not expressed differentially (b), 168 true ESORGs (c) were identified. **(B,C)** *Venn diagram* and heat map revealed that the majority (142 genes; 85%) of ESORGs were downregulated in *var2/flu* in comparison to *flu* upon dark-to-light shift. **(D)** Expression of ^1^O_2_-specific and EX1/EX2-dependent genes *SIB1, WRKY33* and *WRKY40* in *var2/flu* and *flu*. The average values and standard error of the normalized gene expression (reads per million; rpm) of three independent biological samples obtained by RNA-seq analysis were shown. **(E)** Validation of RNA-seq using those selected genes **(D)** by qRT-PCR. Results represent means of three independent biological replicates. *Actin2* was used as a control for normalization.

In earlier studies, the SORGs identified in *flu* mutant (Danon et al., 2006; Laloi et al., 2007; Lee et al., 2007) were employed to address the probable activation of ^1^O_2_ signaling in various mutants and wild-type plants under photooxidative stress conditions (e.g., Ramel et al., 2013). Afterward, the detection of the rapid loss of chloroplast integrity following ^1^O_2_ burst, as evidenced by the appearance of stroma proteins into cytosol (Kim et al., 2012; Chen et al., 2015), prompted us to redefine SORGs as a group of genes that were upregulated prior to the loss of chloroplast integrity (Chen et al., 2015) (399 genes; Supplementary Table S1). In addition, in all of those previous transcriptome data, the confounding effect of dark on the gene expression changes was not excluded. Hence, in the present study, we carried out RNA-seq-based gene expression analysis in 5-day-old seedlings of *flu* and FtsH2-deficient *var2*/*flu*, initially grown under continuous light (LL), and then subjected to 4 hours dark (4hD) followed by re-illumination for 30 (L’30) and 60 min (L’60). Samples harvested at the end of dark were included as additional control to exclude genes upregulated in the dark. As a result, we found that a large proportion (215 genes, 53%) of previously identified SORGs (399) appeared to be upregulated in the dark followed by a gradual repression during re-illumination (**Figure 1A**). These genes were reclassified as dark-induced genes (DIGs) and were excluded from the SORGs, together with 26 more genes that were either not expressed or significantly changed in the expression. The remaining 168 SORGs were thus considered as early ^1^O_2_-responsive genes (hereafter ESORGs) (**Figure 1A** and Supplementary Table S2). The 168 ESORGs include *SIGMA FACTOR BINDING PROTEIN 1* (*SIB1*), *WRKY33* (*WRKY DNA-BINDING PROTEIN 33*), *WRKY40* (*WRKY DNA-BINDING PROTEIN 40*), *ETHYLENE RESPONSE FACTOR 104* (*ERF104*), *MAP KINASE KINASE 9* (*MKK9*), *SENESCENCE ASSOCIATED GENE 20* (*SAG20*), *TOLL/INTERLEUKIN-1 RECEPTOR-LIKE* (*TIR*) and many other stress-responsive genes (**Figure 1A** and Supplementary Table S2). This set of true ESORGs was used for further comparative analysis between *flu* and *var2*/*flu*.

### FtsH2 Is Essential for the Induction of ESORGs

The comparison of transcriptome profiles of *flu* and *var2*/*flu* revealed that approximately 85% of ESORGs (142 out of 168 genes) were repressed in *var2*/*flu* as compared to *flu* (**Figures 1B,C** and Supplementary Table S3). In an earlier study, only three SORGs, namely *SIB1, WRKY33* and *WRKY40*, were shown to be upregulated not only in wild-type seedlings under photoinhibitory condition (Chen et al., 2015) but also in *flu* mutant upon dark-to-light shift in EX1/EX2-dependent manner. Hence, these genes were considered as ^1^O_2_-specific and EX1/EX2-dependent genes (**Figure 1D**). The RNA-seq result was further validated by quantitative real time PCR (qRT-PCR) using these genes (**Figure 1E**). In consistent with the phenotype of *var2*/*flu* seedlings, which appear to be more tolerant to ^1^O_2_ as compared to *flu* mutant seedlings (Wang et al., 2016), the global gene expression levels were less responsive to ^1^O_2_ in *var2*/*flu* seedlings than in *flu* seedlings. The repressed expression of ESORGs following re-illumination and the concurrent attenuation of stress responses suggest that FtsH2 is a positive regulator of EX1-mediated ^1^O_2_ signaling in *flu* mutant.

### Comparative Analysis of β-CC-Induced and EX1-FtsH2-Dependent SORGs

**FIGURE 2:**
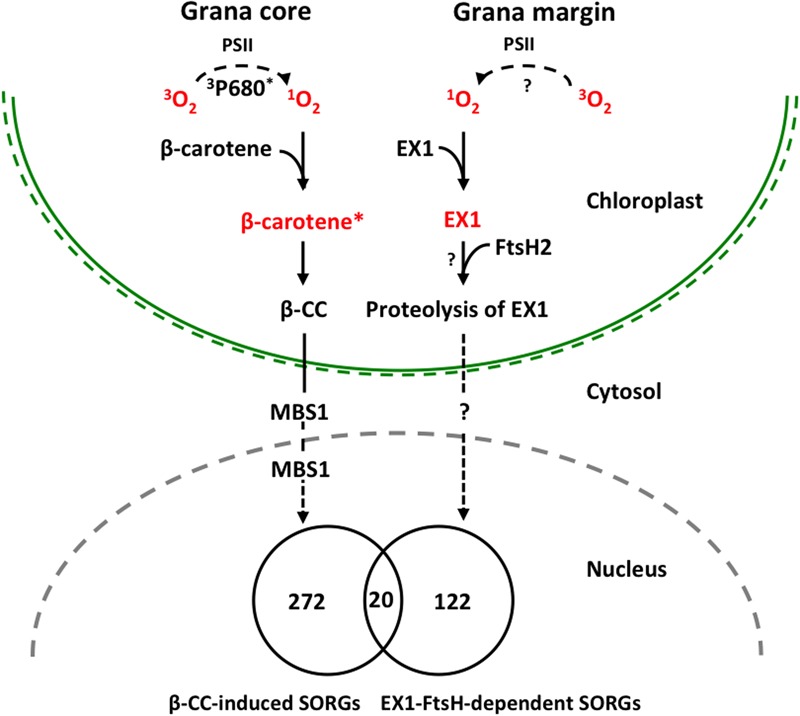
Proposed model illustrating ^1^O_2_-triggered EX1-independent and EX1-dependent chloroplast-to-nucleus retrograde signaling pathways. Under severe light stress, β-carotene from PSII RC enriched in grana core acts as a sensor of ^1^O_2_ generated by the excited triplet P680 chlorophyll (^3^P680^∗^) through the energy transfer from ^3^P680^∗^ to stable oxygen molecule (^3^O2). The subsequent oxidation of β-carotene (β-carotene^∗^) by ^1^O_2_ concomitantly release β-CC, a volatile oxidized derivative, which functions as a messenger involved in the ^1^O_2_ signaling pathway. MBS1 protein, a zinc finger protein located in cytoplasm and nucleus, acts downstream of β-CC. In grana margin, EX1 may sense ^1^O_2_ generated through an as yet unknown process and initiates ^1^O_2_ signaling in an FtsH2-dependent manner. FtsH2-dependent proteolysis of EX1 appears to be essential in the induction of nuclear gene expression changes. β-CC-induced and EX1-FtsH2-dependent SORGs are reasonably distinct as evidenced that these transcriptomes share relatively a very small portion of genes, signifying the coexistence of these two ^1^O_2_ signaling cascades in plants.

Previous studies have established the role of β-CC in ^1^O_2_-triggered acclimation responses (Ramel et al., 2012, 2013), whereas the EX1-mediated ^1^O_2_ signaling has been mainly attributed to PCD in young seedlings and growth inhibition in mature plants (Wagner et al., 2004; Kim et al., 2012). In a very recent study, however, the role of EX1 and EX2 in an acclimation response to high light-induced transcriptional reprogramming has also been demonstrated (Carmody et al., 2016). To get insight into these distinctive cascades, we compared the β-CC-induced SORGs and EX1-FtsH2-dependent ESORGs, with the aim to explain the common and distinct molecular characteristics and enlighten their coexistence. Plants treated with β-CC for 4 h under normal light conditions exhibited a rapid reprogramming of nuclear gene expression, resulting in the altered expression of around 380 genes with at least twofold up- or down-regulation (Ramel et al., 2012). Among these 380 genes, the upregulated genes (292) are considered as β-CC-induced SORGs (Supplementary Table S4). Comparative analysis revealed that β-CC-induced (292) and EX1-FtsH2-dependent (142) genes shared only a small number of genes (20) (**Figure 2**). The shared genes include above-mentioned ^1^O_2_-responsive genes, such as *WRKY33, WRKY40*, and *SIB1*. However, the expressions of these genes were comparatively higher in *flu* mutant (Supplementary Table S5). Taken together, these results further support the coexistence of the two independent ^1^O_2_-signalings in Arabidopsis. Considering that ^1^O_2_-specific and EX1/EX2-dependent genes including *WRKY33, WRKY40* and *SIB1*, appear to be upregulated in response to β-CC, we cannot rule out the possibility that these two signaling cascades may share a certain downstream component to regulate those shared genes.

## Summary and Future Directions

In summary, our previous study revealed the important role of ATP-dependent FtsH metalloprotease, assembled in thylakoid membrane and functioning in PSII repair cycle, in the induction of EX1-mediated stress response upon ^1^O_2_ generation in chloroplast. The FtsH protease is co-localized with EX1 in grana margin where the PSII repair process undergoes, and it coordinates the retrograde signaling by promoting the proteolytic degradation of EX1 proteins through a yet unknown mechanism (**Figure 2**). FtsH-dependent EX1 degradation was found to occur upon ^1^O_2_ burst in chloroplast, which possibly triggers the signaling. In agreement, inactivation of FtsH2, a subunit of the FtsH protease complex, appears to considerably compromise the proteolysis of EX1 and the concurrent ^1^O_2_-triggered PCD in Arabidopsis *flu* mutant. Furthermore, the loss of FtsH2 in *flu* resulted in the repression of the majority of SORGs. β-CC-induced and EX1-FtsH2-dependent SORGs are largely distinct, sharing relatively a very small portion of genes. These results provide compelling evidence that chloroplasts may operate two distinctive ^1^O_2_ signaling: one operated in grana core by β-carotene and one in grana margin by the coordination of EX1 and FtsH2 (**Figure 2**). Based on this foundation, further investigation on EX1-FtsH2-dependent signaling is needed in order answer questions such as (i) what is the source of ^1^O_2_ in the grana margin? (ii) how EX1 senses and mediates this signaling in coordination with FtsH2? (iii) what is the genuine signaling molecule that is generated upon EX1 proteolysis? (**Figure 2**). Furthermore, the reasons for the coexistence of two distinctive ^1^O_2_-triggered retrograde signaling pathways in plants are still unknown, which probably opens new perspectives of research in this direction.

## Materials and Methods

### Plant Materials, Growth Conditions, and RNA Isolation

Seedlings of *flu* and *var2*/*flu* mutant lines (Wang et al., 2016), all in the Columbia-0 (Col-0) background, were grown under continuous light (40 μmol photons m^-2^ s^-1^) at 20 ± 1°C on 0.6% agar plates containing ½ MS medium and 1xGamborg vitamins. Total RNA was isolated using RNA easy Plant mini kit (Qiagen, Germany). DNase I (Qiagen, Germany) treatment was performed to remove potential DNA contamination. RNA was examined by a Nano Photometer spectrophotometer (Implen, Westlake Village, CA, United States) for RNA purity. Qubit RNA Assay Kit in Qubit 2.0 Flurometer (Life Technologies, Foster City, CA, United States) was used to measure RNA concentration, and RNA Nano 6000 Assay Kit of the Bioanalyzer 2100 system (Agilent Technologies, Santa Clara, CA, United States) was used to evaluate RNA integrity. Only RNA samples that passed the quality control were used for RNA-Seq analyses. Three biological replicates were subject to RNA-Seq based gene expression analysis for each genotype/treatment.

### RNA-seq Library Construction, Sequencing, and Analysis

RNA-seq libraries were constructed using the NEBNext Ultra Directional RNA Library Prep Kit for Illumina (NEB, Ipswich, MA, United States) following manufacturer’s instructions. RNA-seq libraries were sequenced on an Illumina HiSeq 2500 platform to generate 100 bp paired-end reads as described previously (Zhao et al., 2016). Lowly expressed genes were removed and genes with an expression level of at least 1 transcripts per million (TPM) in at least three samples were selected for differential expression analysis. The R package edgeR, which uses counts per gene in different samples from HTSeq-count as input and performs data normalization using the trimmed mean of M-values (TMM) method, was used to identify differentially expressed genes (DEGs). The genes with at least twofold change in expression and the false discovery rate (FDR) of less than 0.05 were considered to be differentially expressed. Gene expression levels were normalized to TPM according to the total number of mapped clean reads in each of library. The log2 values of normalized expression levels were used to build expression matrix and subsequent clustering and visualization was conducted using Multi-Experiment Viewer (MeV 4.9.0).

### qRT-PCR Analysis

Quantitative real time PCR assays were performed with samples that were collected independently of samples used for RNA-Seq analysis. Three biological replicates were used for each genotype/treatment. RNAs were treated with RQ1 RNase-Free DNase (Promega) and reverse-transcribed using Improm II reverse transcriptase (Promega) and oligo(dT)15 primer (Promega) according to the manufacturer’s recommendations. The qRT-PCR was performed by using the QuantStudio^TM^ 6 Flex Real-Time PCR System (Applied Biosystems) and iTaq Universal SYBR Green PCR master mix (Bio-Rad). Relative transcript levels were calculated by the comparative delta-Ct method and normalized to the *ACT2* (At3g18780) gene transcript level. The primers used in this study were designed by Primer Express Software for Real-time PCR, Version 3.0 (Applied Biosystems) and the primer sequences are included in Supplementary Table S6.

## Author Contributions

VD, KL, JD, and CK planned and designed the research; VD, JD, and KL performed research; SL and VD analyzed RNA-seq data; and VD, RL, and CK wrote the paper.

## Conflict of Interest Statement

The authors declare that the research was conducted in the absence of any commercial or financial relationships that could be construed as a potential conflict of interest.
