# Supplementary material for: FtsH2-Dependent Proteolysis of EXECUTER1 Is Essential in Mediating Singlet Oxygen-Triggered Retrograde Signaling in Arabidopsis thaliana
Source: Front Plant Sci. 2017 Jun 29;8:1145. doi: 10.3389/fpls.2017.01145 (PMC5489589; doi:10.3389/fpls.2017.01145)
Supplement: Supplementary file 6 [file Table_6.DOCX]

**Table S6: List of Primers used for qRT-PCR analysis.**

| **Gene Symbols** | **Locus** | **Primer (5′-3′)** |
| --- | --- | --- |
| SIB1 F | At3g56710 | CGACTTTTCTCACCACGACA |
| SIB1 R |  | TCGGAGGAAGGGGAATAGAT |
| WRKY 33 F | At2g38470 | GAAACAAATGGTGGGAATGG |
| WRKY 33 R |  | TGTCGTGTGATGCTCTCTCC |
| WRKY 40 F | At1g80840 | GTGGAGGATCAGTCCGTGTT |
| WRKY 40 R |  | TCTGAACTTGGGGAAAATCG |
| ACT2 F | At3g18780 | GGCTCCTCTTAACCCAAAGG |
| ACT2 R |  | CAGTAAGGTCACGTCCAGCA |
